# Nature limits filarial transmission

**DOI:** 10.1186/1756-3305-1-13

**Published:** 2008-05-23

**Authors:** Goutam Chandra

**Affiliations:** 1 Mosquito Research Unit, Department of Zoology, The University of Burdwan, West Bengal, India

## Abstract

Lymphatic filariasis, caused by *Wuchereria bancrofti, Brugia malayi *and *B. timori *is a public health problem of considerable magnitude of the tropics and subtropics. Presently 1.3 billion people are at risk of lymphatic filariasis (LF) infection and about 120 million people are affected in 83 countries. In this context it is worth mentioning that 'nature' itself limits filarial transmission to a great extent in a number of ways such as by reducing vector populations, parasitic load and many other bearings. Possibilities to utilize these bearings of natural control of filariasis should be searched and if manipulations on nature, like indiscriminate urbanization and deforestation, creating sites favourable for the breeding of filarial vectors and unsanitary conditions, water pollution with organic matters etc., are reduced below the threshold level, we will be highly benefited. Understandings of the factors related to natural phenomena of control of filariasis narrated in this article may help to adopt effective control strategies.

## Introduction

Lymphatic filariasis, caused by *Wuchereria bancrofti, Brugia malayi *and *B. timori *is a major public health problem of the tropics and subtropics. According to the Expert Committee on Filariasis, 905 million people were at risk of lymphatic filariasis with 90.2 millions of victims worldwide in 1984 [1] and the figures were 751.4 millions and 78.6 million in 1992 [2]. Control Programmes, with DEC and/or Ivermectin treatment [3-7] of the human host and vector control by different means [8-13] have been undertaken. At present, world wide 1.3 billion people are at risk of lymphatic filariasis (LF) infection and about 120 million people are affected in 83 countries [14]. Amongst them 45.5 million live in the Indian subcontinent and 40 million in Sub-Saharan Africa [15]. In this alarming situation, it is my aim to highlight that 'nature' itself limits filarial transmission to a great extent through different means such as by reducing vector populations, parasitic load etc otherwise the situation might have been graver. Any or more of the natural phenomena narrated below may be utilised judiciously to secure better control methods of filariasis besides methods involved in the Global Program for Elimination of Lymphatic Filariasis (GPELF) launched in 1999 [16].

The ecological factors like temperature and humidity play a significant role in filariasis transmission [17]. It is generally recognized that considerable temperature and high humidity are necessary for the survival of most vector insects but the effects of those two factors may be more vital on the development of *Wuchereria *larvae in its vector [18]. Bancroft [19] noticed that the microfilariae required 16–17 days in the mosquito vector to reach the infective stage and his view was corroborated by Low and James [20,21]. Under experimental conditions, development of lymphatic filarial parasites in the mosquito takes two weeks at 27°C and 90% humidity [22]. The period of larval development varies with season [23]. At high temperature and moisture the complete cycle occupies 10–14 days but it is retarded to 6 weeks by cold [17].

### Lack of synchronization between transmission season and the period of higher vector density

Experiments on the filarial vector *Culex quinquefasciatus *revealed that its density was found to be significantly lower (p < 0.05) in the rainy season in comparison to dry seasons (summer and winter) in different endemic areas of the tropics [24-38] because their breeding places become flooded during the monsoon. On the other hand, the hot months of the rainy season and sometimes summer were found to be the high time for filarial transmission in most of the endemic areas, established by the highest infection and infectivity rates (with filarial parasites) of the vector in nature [31,35,39-42], the shortest developmental period of the parasite in the vector [23] and highest transmission potential [43,44] during this period of the year. The rainy season provides optimum conditions to raise the vector efficiency index (VEI) to its peak (VEI is based on rapid parasitic development, proper nursing and low parasitic damage or death) [23].

So, there is a lack of synchronization between two important factors like transmission season and the period of highest vector density, which limits transmission and keeps it at a low level.

### A sharp fall of parasite load in the process of transmission

When parasite load was examined, a sharp reduction was noticed between the load of microfilariae per infected mosquito and the load of third stage infective larvae (L_3_) per infected mosquito (reduction is significant ; p < 0.05) in both urban and rural micro environments [43,45]. In Fiji, Symes [46] also obtained similar results. It indicates that all the microfilariae that enter into the gastrointestinal tract of mosquito cannot survive to develop into L_3 _stage. Bryan *et al *and McGreevy *et al *[47,48] reported that sometimes microfilariae are damaged by the buccopharyngeal armature of the mosquito during ingestion. Rise or fall of temperature and fall of humidity caused deformity and degeneration of a large number of filarial parasites in the mosquito body [23,35]

Mosquitoes limit the number of migrating microfilariae by rapidly excreting them. Wharton [49] found an average of more than 100 microfilariae to be ejected in droplet from the anus of *An. barbirostris *fed on a cat infected with *Brugia pahangi*. Similar results were obtained with *W. bancrofti *in *Cx. quinquefasciatus *[50].

Hu [51] and Chandra *et al *[23] noted that all the microfilariae failed to escape from the midgut of the infected mosquito due to low temperature in winter in Shanghai, China and West Bengal, India respectively.

Sutherland *et al *[52], Yamamoto *et al *[53] and Christensen *et al *[54] reported on the defence mechanism and defence reaction of mosquito vectors to filarial worms. The growth of a high percentage of parasites is arrested in their sites of development of the vector body due to melanization and encapsulation. The antihemostatic factors present in saliva allow mosquitoes to blood-feed efficiently, but different mosquito species can differ markedly in blood feeding potency [55]. Further, the fluid consistency of ingested blood usually varies in different mosquitoes. The coagulation of ingested blood within the mid-gut also inhibits ingested pathogens from migrating out of the gut to reach the final site of development. This potential barrier to pathogen development varies significantly depending on the length of time a pathogen spends within the mid-gut. But evidently the consistency of ingested blood greatly influences both the prevalence and intensity of infection for all mosquito-borne parasites [56,57].

Kobayashi et al studied filariasis [58] and analysed the refractory mechanisms of the mosquito *Aedes aegypti *to the filarial larvae *Brugia malayi *by means of parabiotic twinning.

The fate of larvae after leaving the proboscis is not known for *W. bancrofti*, but experimental data for *Brugia pahangi *[59,60] revealed that during a single complete feeding 32% only of the escaping larvae succeeded in penetrating the tissues of experimental hosts. Similar results were obtained when the mosquito had a partial blood meal (31.3%) or made multiple attempts to feed (38,1%), when a single feeding attempt was unsuccessful, 10.2% of the escaping larvae succeeded in penetrating the tissues.

The most important factor in estimating the extent to which accumulation is possible is the rate of survival of immature parasites in the human. Again, data for *W. bancrofti *are not available, but Edeson & Buckley [61] obtained a survival to maturity of 0.13 for *Brugia malayi *in experimental animals, assuming a constant death rate during this immature period of 2 1/2 months, the daily mortality can be calculated as

0.13 = e^-75d^

When d is the instantaneous death rate per day and e is the base of natural logarithms. From the equation, e = 0.027/day, and *W. bancrofti *is known from unpublished WHO information to have a minimal prepatent period of 8 month and 4 days. Thus if we apply the calculated death rate, the proportion of the larvae surviving would be 0.00147 [61,62].

So, a sharp reduction in parasite load during parasitic development in the vector, revealed from the above literature, limits transmission and keeps it at a low level.

### Vector mortality

Numbers of *Cx*. *quinquefasciatus *carrying microfilariae, first stage, second stage and third stage larvae of *W. bancrofti *in nature gradually decreased in both urban and rural areas [43,45]. It is an indication that all the mosquitoes (initially infected with microfilariae) cannot survive the period required for the development of microfilariae into third stage infective larvae. Different investigators recorded varied daily mortality rate of *Cx. quinquefasciatus *from 14% to as much as 47% in different parts of the world [35,63-70]. Almost all the authors, who worked on the vector infection and infectivity of different filarial vectors of different parts of the world [43,45,71] recorded significantly higher (p < 0.05) infection rate than that of infectivity rate of a particular endemic area, which is another indication of substantive vector mortality in a given period.

When presumptive mortality rate [72] of *Cx. quinquefasciatus *(collected from both urban and rural areas) between two successive gonotrophic cycles was determined, a high percentage of mortality of vector population was observed between two successive gonotrophic cycles. A high mortality between two successive gonotrophic cycles caused considerable reduction of vector as well as parasite population naturally.

Most of the mosquitoes carrying microfilariae were found to be nulliparous (yet to lay first batches of eggs) i.e. took microfilariae during their first blood meal when parity status was determined by Polovodova's method [73]. Pentaparous mosquito containing microfilariae proves that they may also be infected during their sixth blood meal but with the increase of age of mosquitoes, new infection with microfilariae decreased [74]. So, natural vector mortality during the period required for the development of microfilariae into third stage infective larvae  is indicative of reduction in transmission.

### Other bearings of natural control

Moreover, *Wuchereria *spp., which causes the major global burden (106.2 million out of 119.1 million), is not a zoonotic parasite i.e. man is the only known definitive host and there is no other reservoir. Except for a mosquito bite, there is no other mode of transmission. Though congenital microfilaremia has been reported [75,76], it is of no significance. Microfilariae transmitted by blood transfusion may survive and circulate up to very limited number of days and do not develop into adult worms [17]. It can be shown in principle that there are critical densities of host and vector below which the parasite population cannot be maintained and that these critical densities are most important for parasites in which the sexes are separate [77]. This is true for all nematodes, as their sexes are separate, and here specifically for *Wuchereria *and *Brugia*. Multiple bites by infective mosquitoes are required for effective transmission. The WHO Filariasis Research Unit in Rangoon estimated that an average of around 15,500 bites by infective mosquitoes is necessary to produce 1 case of microfilaremia [62]. The major vector of nocturnally periodic *Wuchereria (Cx. quinquefasciatus) *breeds only in man-made polluted water and *Mansonioides*, vectors of *Brugia *breed in water bodies infested with certain preferred weeds (*Pistia, Eichornia*, *Lemna*) only [78]. At the same time they cannot oviposit in water bodies infested with ferns like *Azolla, Salvinia *etc [78]. So, vast water bodies, which are free of, preferred weeds or infested with *Azolla *or *Salvinia *do not favour breeding of *Brugia *vectors.

According to Dreyer *et al *factors associated with adult worm longevity are unknown [79]. They concluded that survival of adult *W. bancrofti *is inversely associated with transmission intensity.

Snow and Michael provided clear evidence for the existence of microfilarial density-dependence in the process of transmission for all the three major bancroftian filariasis transmitting mosquito vectors [80]. The regulation of mf uptake varied significantly between the vector genera, being weakest in *Culex*, stronger in *Aedes *and most severe and occurring at significantly lower human mf loads in *Anopheles *mosquitoes. It indicates lower intensities of transmission in the vast endemic areas where *Culex *acts as vector.

Dissanayake showed that development of adenolymphangitis and lymphoedema was strongly associated with amicrofilaraemic infection [81]. In contrast, microfilaraemic individuals are more likely to remain microfilaraemic without developing clinical lymphatic disease. It is concluded that asymptomatic microfilaraemia and amicrofilaraemic clinical disease are independent outcomes of *W. bancrofti *infection and are not sequential events of progressive infection. This indicates that most of the microfilaraemics do not develop symptoms and morbidity. Therefore, all these mechanisms have a natural bearing in limiting transmission.

## Conclusion

Collectively, 'nature' plays a great role in limiting filarial transmission throughout the way of its transmission dynamics. Those people who are close to nature, such as, tribal peoples (they live in small rural set up isolated from urban areas and devoid of indiscriminate urbanization) in the developing countries are safe to a large extent from the menace of filariasis partly due to their cultural practices (they keep their courtyards and surroundings very neat and clean, without any ditches or mosquitogenic sources) [82-84]. So, we can avail ourselves the blessings of the bearing of natural control of filariasis to a greater extent if manipulations on nature are reduced. One basic point of Millennium Development Goal [85] i,e. 'Respect to nature' is relevant in this context.

Filarial transmission can be reduced through man-made interventions that include source reduction i.e. reduction of mosquito larval habitats, use of natural predators, application of larvicides and adulticides of biological and chemical origins. Using these methods alone or in combination has proved helpful in regulating vector population, but to a limited extent. Emphasizing biological resources and products that would keep the vector population below the threshold level in framing strategies for regulating filarial transmission is highly required. Biological resources or products will not interfere with the natural regulation of transmission. The target points for man-made control of filariasis during transmission are restricted to vector mosquitoes, while nature imparts a regulatory effect on both the vectors and the filarial worms in the vectors. Therefore, the processes limiting filarial transmission naturally, needs to be given priority to frame effective control strategies. For instance, a record or a monitoring of the abundance of the vector population and its seasonality can be used as a background to determine the time of control operation.

As filarial vector density increases in the dry months in many endemic areas [24-38], larvicides and adulticides (preferably of biological origin) may be applied just before dry season. At the same time larval predators may also be introduced in the larval habitats where such predators are absent at the onset of dry season. On the other hand, elimination of aquatic weeds that facilitate breeding of *Mansonioides *mosquitoes [78] mechanically or by using weedivorous fishes be done when they grow well in the wet months.

It is known that reappearance of microfilariae or recurrence of microfilaremia occurs after certain period of time in certain percentage of single dose DEC treated microfilaremics [45,86] and low-density microfilaremia has an important role in transmitting filariasis [87]. Thus to desynchronise the period of recurrence in microfilaremia and the period of effective transmission and also to reduce overall transmission, yearly single dose mass DEC treatment (under GPELF) can be given just before monsoon when transmission occurs very effectively [23,31,35,39-44]. Proper measures can be adopted to avoid bites of *Cx. quinquefasciatus *during the peak period of filarial transmission in a 24-hour period i.e. the 3^rd ^quadrant of night (12 mid night to 3 a.m.) [88]. Other target points are to reduce indiscriminate urbanization and deforestation, creating mosquitogenic sites and unsanitary conditions, water pollution with organic matters etc. below the threshold level.
